# RACIAL DISPARITY IN THE START OF HOME HEALTHCARE IN HIGH-RISK ADRD PATIENTS BY THE QUALITY OF HOME HEALTH

**DOI:** 10.1093/geroni/igac059.1544

**Published:** 2022-12-20

**Authors:** Indrakshi Roy

**Affiliations:** Northern Arizona University, FLAGSTAFF, Arizona, United States

## Abstract

Improving the quality and timely access of home health care is a new quality measure and particularly crucial in high-risk ADRD adults following hospitalization. However, a significant portion of older patients waits longer than 2 days to receive home healthcare. In this study, we examine how the quality of home health agency and race are associated with the delay in care among ADRD patients receiving home healthcare and how this delay mitigates the risk of rehospitalization. We find that Black patients in low rated home health agencies have 28% higher odds of delay in care compared to White patients in high rated home health agencies (Odds ratio (95% CI) =1.28 (1.21 - 1.36)). Timely initiation of home health care also reduces the risk of rehospitalization in minority older adults with ADRD.

